# Challenges of Co–Cr Alloy Additive Manufacturing Methods in Dentistry—The Current State of Knowledge (Systematic Review)

**DOI:** 10.3390/ma13163524

**Published:** 2020-08-10

**Authors:** Bartłomiej Konieczny, Agata Szczesio-Wlodarczyk, Jerzy Sokolowski, Kinga Bociong

**Affiliations:** 1 University Laboratory of Materials Research, Medical University of Lodz, ul. Pomorska 251, 92-213 Lodz, Poland; agata.szczesio@umed.lodz.pl (A.S.-W.); kinga.bociong@umed.lodz.pl (K.B.); 2 Department of General Dentistry, Medical University of Lodz, ul. Pomorska 251, 92-213 Lodz, Poland; jerzy.sokolowski@umed.lodz.pl

**Keywords:** powder bed fusion, selective laser sintering, selective laser melting, electron beam melting, process parameters, stress relieving, heat treatment, cobalt–chromium, dentistry

## Abstract

Complex dental components which are individually tailored to the patient can be obtained due to new additive manufacturing technology. This paper reviews the metallic powders used in dental applications, the fabrication process (build orientation, process parameters) and post-processing processes (stress relieving, surface finishing). A review of the literature was performed using PubMed, ScienceDirect, Mendeley and Google Scholar. Over eighty articles were selected based on relevance to this review. This paper attempts to include the latest research from 2010 until 2020, however, older manuscripts (10 articles) were also selected. Over 1200 records were identified through the search; these were screened for title and/or summary. Over eighty articles were selected based on relevance to this review. In order to obtain a product which can be used in clinical applications, the appropriate manufacturing parameters should be selected. A discussion was made on optimal selective laser melting (SLM) parameters in dentistry. In addition, this paper includes a critical review of applied thermal treatment methods for Co–Cr alloys used in dentistry.

## 1. Introduction

CoCr-based alloys are commonly used in dentistry because of their excellent corrosion resistance and outstanding mechanical properties, such as high stiffness. Since its development in 1907, lost wax casting is still the dominant method of dental metal processing [1]. Unfortunately, this technique has some limitations, one of which is the fact that metal shrinks during its transition from the liquid to the solid phase, and this shrinkage should be taken into account when preparing the part for casting. Additionally, pores and other defects are usually present in the structure of the cast element [2,3], the process is time-consuming and requires certain skills for the operators [4], and CoCr alloys are difficult to treat and process because of their high hardness [5].

Nowadays, metallic materials are processed by computer-aided design and computer-aided manufacturing (CAD–CAM) (Table 1) that allows 3D structures to be produced based on data (appropriate conversed–segmented) regarding individual organs, bones or blood vessels obtained from medical imaging devices such as magnetic resonance imaging (MRI), computed tomography (CT), cone beam tomography (CBCT) or ultrasound (USG). Segmentation and analysis software is available as open source versions (e.g., InVesalius or 3D Slicer) and as a paid version with extended functionality (Materialize Mimics, Amira or Dolphin 3d). Three-dimensional models can also be obtained using 3D scanning; this is the most common type of solution used in Dentistry (i.e., in prosthetics and orthodontics), using optical scanners employing photogrammetry or scanning with structured light. The 3D data obtained in this way are used for the design of prosthetic restorations, orthodontic appliances, surgical templates or individualized implants using dedicated CAD software [6,7,8,9]. 

CAD–CAM technology encompasses two main categories: subtractive manufacturing processes like milling, and additive manufacturing (AM) processes like powder bed fusion [10,11]. Milling uses tools (saws, lathes, grinders, drill presses) to mechanically cut a block to a desired geometry and this process is controlled by software. Compared to the casting method, this technique can reduce flaws and pores because the blanks are made under high industrial standards [12,13]. However, the process is associated with a greater waste of materials and some limitations in complex projects compared to casting and AM [10]. AM technologies, more popularly known as ‘three-dimensional (3D) printing’, build physical objects in a single stage directly from their computer-aided design. With this technology, the products are formed through the addition of the materials layer by layer, based on sliced data from the 3D design [14,15]. Therefore, this method is used for producing complex products and has opened new possibilities in dentistry [16]. AM can use polymers, ceramics, metal alloys and composites. New approaches are focused on the development of biological ink [15,17]. Some common uses of AM techniques in dentistry are stereolithography (SLA), fused deposition modeling (FDM), powder bed fusion (PBF) and ink-jet printing (IJP) [18]. Of these, powder bed fusion (PBF) is commonly employed for metal processing in dentistry [19,20]. Powder bed fusion uses a heat source to consolidate metallic powder to form a 3D object (layer by layer), according to CAD. This techniques offer cost-effective customization and reduced assembly [21]. 

## 2. Materials and Methods 

An electronic search was performed in the databases PubMed, ScienceDirect, Mendeley and Google Scholar. A manual search of citations from relevant articles was also conducted. The following keywords were used: powder bed fusion (or PBF), selective laser sintering (or SLS), selective laser melting (or SLM), electron beam melting (or EBM), inclination angle, stress relieving, heat treatment, cobalt–chromium (or Co–Cr), dental. This paper attempts to include the latest research from the last 10 years (2010–2020), however, older manuscripts were also selected. Over 1200 records were identified through the search; these were screened for title and/or summary. Over eighty articles were selected based on their relevance to this review. The exclusion criteria included non-dental (or non-medical) applications and the articles on 3D printing of metal alloys other than Co–Cr. The results were based on a descriptive analysis of the techniques and materials of additive manufacturing, fabrication process and post-processing strategies.

## 3. Powder Bed Fusion (PBF)

Powder bed fusion can be subdivided into three methods: selective laser sintering (SLS), selective laser melting (SLM) and electron beam melting (EBM). In SLS, metal powder compacts are transformed into coherent solids at temperatures below their melting point. In SLM and EBM, the metal powder is fully melted, however, the two methods use different ways to achieve their melting point [23].

### 3.1. Selective Laser Sintering (SLS)

Carl Deckard and Joe Beaman developed and patented in 1989 selective laser sintering (SLS) technology [24]. In this method, a high-power laser is focused onto a thin layer of metal powder. The layer is heated, and next bonding processes between metal particles is started. This step requires the transport of material from inside the powder to points and areas where particles are in contact with each other. There are five different transport mechanisms possible: volume diffusion, grain-boundary diffusion, surface diffusion, viscous or plastic flow. Elements made by SLS technique are characterized by high porosity. It is crucial to correctly select the parameters of the SLS process (temperature, time, geometrical structure of the powder particles, composition of the powder mix, density of the powder compact, composition of the protective atmosphere in the sintering furnace) [25]. Unfortunately, the complete elimination of porosities is not possible due to the partial melting and sintering caused by the melting point not being reached [26].

### 3.2. Selective Laser Melting (SLM)

It is possible to melt metal powder using powerful, high-quality lasers. In 1995, commercial machines using SLM technology were launched on the market. Selective laser melting printers use CO_2_ lasers or fiber lasers (Nd:YAG or Yb:YAG) [27]. The laser beam is focused onto the powder, and the energy supplied by the laser is able to melt it. The melting process can be adjusted by varying the wavelength, laser source and power. The resulting products are characterized by a lower occurrence of blisters and a better superficial finish than those made by SLS. Unfortunately, high internal stresses are found in materials, caused by the thermal gradients induced during manufacturing. To reduce these stresses, additional heat treatment is required [19,28,29]. Selective laser melting technology is the most popular approach to metal processing in Dentistry (Table 1).

### 3.3. Electron Beam Melting (EBM)

This technology is very similar to SLM. However, EBM technology uses a focused electron beam in a vacuum environment to melt the layers of powder. During EBM processing, a tungsten filament is heated, and this gives off electrons which are accelerated into a beam by two magnetic coils. The beam strikes the metal powder, which is melted by the transmission of kinetic energy [30,31].

## 4. Metallurgy

The most commonly used alloys for PBF in dental applications are cobalt–chromium (Co–Cr) and Titanium (Ti). Other metals are also added to improve the strength properties of the material. Co–Cr-based alloys are the hardest known biocompatible materials that are commonly used in medicine and dentistry. They demonstrate good fatigue resistance, tensile strength, elasticity or corrosion resistance, and are suitable for use in medical implants, partial skeletal denture frameworks and for crown and bridge substructure restorations in dentistry [31,32]. Chromium forms a solid solution with cobalt and increases corrosion resistance by surface passivation; however, a chromium content greater than 30% results in difficulty in casting and the formation of a brittle σ phase. Molybdenum (Mo) and wolfram (W) cause solid solution strengthening, and the formation of M6C, MC type carbides and Co3M intermetallic phase (M represents a metal element). Molybdenum affects the grain size and reduces the susceptibility to pitting corrosion. Silicon (Si) and manganese (Mn) are added to improve the alloy’s fluidity, while niobium (Nb) affects the solution strengthening, intermetallic phase formation and MC type carbides. The cobalt-based alloys used in dentistry have a low carbon (C) content. The addition of carbon has a very significant impact on mechanical properties, and especially on plasticity, even in small quantities. High strength and creep resistance (resistance to plastic flow) are obtained at a carbon content of 0.3–1.0%. Due to the presence of carbide-forming additives (with a significant carbon content), carbides with a complex structure e.g., M23C6 type, are formed in interdendritic spaces during the heat treatment [33,34].

Powder compositions and mechanical properties are summarized in Table 2 and Table 3. Differences can be seen between Co–Cr alloys. Only SLM technology is used to manufacture Co–Cr dental prosthetics [19]. 

## 5. Fabrication Process

The properties of the final product depend on a number of factors. The key element is the choice of powder; however, the optimization of the process parameters is equally important. The most important factors include the development of a model in a computer program, the determination of its build orientation and the selection of machine parameters [18,29,35,36,37]. 

### 5.1. Build Orientation

The building orientation is generally defined as the acute angle between the longitudinal axis of a given sample and the vertical axis of the building platform. Microstructure, texture and residual stress concentration are dependent on the building orientation [38]. The thermal fluctuations occurring during the manufacturing process have the greatest effect on the orientations of the formed grains: in general, the grain grows from a cooler (building platform) to a warmer region (top surface) [39,40].

**Figure 1:**
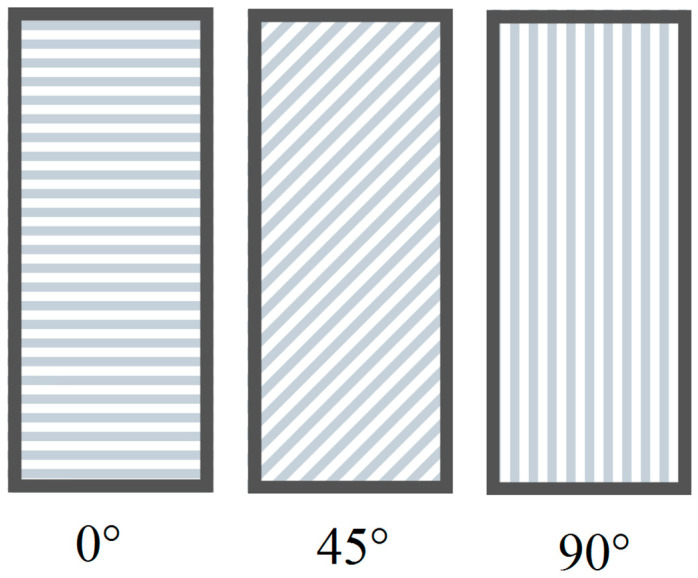
Scheme of the fabricated layers in the SLM samples according to the different building orientations—0°, 45° or 90°.

Selective laser melting manufactured parts are characterized by anisotropic γ-phase, i.e., face-centered cubic (fcc) and ε-phase, i.e., hexagonal close-packed (hcp). The Fcc phase is dominant [41]. Additionally, precipitates were observed to align along the build direction [42]. The anisotropic structure influences the mechanical properties of SLM products. Figure 1 presents a scheme of fabricated layers in SLM samples according to different building orientations. At 45° and 0°, there are more layers, which result in more molten pool boundaries. Fractures in SLM parts can be initiated at the molten pool boundaries and cracks may be observed along the boundaries of tensile fracture extensions. For samples built at 0° orientations, the fracture surface is almost parallel to the molten pool boundaries and cracks may easily propagate along them. Hence, molten pool boundaries appear to have some impact on the anisotropy behavior of SLM builds [43]. 

Materials prepared by PBF exhibited comparable (or even higher) mechanical properties (yield strength, tensile strength) as those prepared by casting. Higher yield strength and lower elongation were observed at 45° or 90° building orientations [41,44]. Samples built at 0° and 45° had lower fatigue strength than the cast samples, but the samples manufactured at 90° have the highest fatigue strength. These properties can be affected by anisotropic microstructure, crystal orientation, surface roughness and residual stress [43,45]. The fatigue strength of metal AM parts can be improved through post-heat treatment [5]. The anisotropy in PBF parts may affect clinical performance. It is essential to apply a proper post-processing treatment to ensure the reliable usage of manufactured products. 

### 5.2. Process Parameters

A layer-by-layer manufacturing process results in complex, time-dependent temperature profiles. Each metallic layer may repeatedly transform between phases (e.g., α → β) and states (from solid to liquid). Such frequent thermal cycling effects the microstructure of the material [46]. Temperature profiles are dependent upon a number of variables, including the AM equipment, the time between passes, and the size of the part being fabricated.

The quality and effects of the metal SLM process is influenced by laser power, scanning speed, laser beam size and layer thickness; these are responsible for the melting energy and penetration depth of alloy powder, which translates into the density of the melted material [47]. As the laser power increases, the amount of powder melted increases per unit of time. Although the width and depth of the molten pool increase, they decrease when we increase the speed of the laser beam feed, while maintaining the same power. Scan line spacing (scanning pitch) i.e., the distance between two adjacent laser scanning trajectories, also influences the characteristics of the molten pool [32,48]. A large scanning pitch may result in a large number of voids, and lowered density, while a small pitch may cause the formation of a coarse grain structure, which affects the performance of the product [32]. Energy input, calculated as P/V, where P is laser power and V scanning speed, is also a determining factor. When the energy input is below 0.36 J/s, the density of the products increases with an increase in energy input. With an increase in energy input, metal powders tend to be completely melted and a full metallurgical bond is achieved between particles. Above 0.36 J/s, the density begins to reduce due to a vaporization of melt pools [49]. 

These process parameters have a significant impact on product performance [50]. The tensile strength and yield strength of the sample increase as the laser power increases and the scanning pitch decreases. However, increasing the scanning speed deceases the tensile strength and yield strength. To achieve the best properties of cobalt–chromium alloys in the SLM process, the optimal parameters seem to be a laser power of 160 W, a scanning speed of 1100 mm/s, and a scan line spacing of 0.05 mm [32].

Laser energy density (LED) or volume energy is an established characteristic, which combines four major process parameters, namely laser power P (W), scanning velocity v (mm s^−1^), distance between two consequent laser scans h (mm) and layer thickness d (mm) (Equation (1)):

(1) $$LED=\frac{P}{v\cdot h\cdot d}\;\left[\frac{J}{mm^{3}}\right]$$

This metric correlates well with the hardness, microstructure and surface morphology. LED values of 150–200 J mm^−3^ are optimal for obtaining high quality Co_28_Cr_6_Mo SLM parts [51].

## 6. Post-Processing Strategies

After the fabrication process, there are a few steps required to obtain the final product. Unprocessed loose powder particles and needless support structures are removed. Following this, the object is prepared for heat treatment. Final processes like surface finishing and sterilization are often used [27].

### 6.1. Stress Relieving

In SLM, a high gradient of temperature and build orientation have a profound influence on microstructures, textures, roughness and residual stress occurrence. As a result, the fabricated object has anisotropic properties dependent on build-up orientation [52,53]. Due to the complex structure of dental prosthesis and various stress directions during mastication, anisotropy and residual stress should be eliminated. Post-processing heat treatment is considered necessary to ensure the reliable use of PFB-built parts in practical applications. The main phases of Co–Cr–Mo alloys are the γ-phase, i.e., the face-centered cubic (fcc) and ε-phase, i.e., hexagonal close-packed (hcp). The γ-phase is stable at a high temperature and the ε-phase at low temperature. The hcp structure is formed while the alloy is cooled from high temperatures. The phase diagram for the Co–Cr alloys shows that γ → ε transformation takes place around 900 °C. The differences in temperature may be due to a different elemental composition [54,55]. 

Table 4 presents the main conditions of heat treatment for PFB-built parts.

It was shown that the heat treatment up to 1050 °C for six hours was insufficient to eliminate anisotropy and residual stress. However, the dendritic structures and number of sub-grains decreased while increasing the heat-treatment temperature (from 750 °C to 1050 °C) compared with the as-built samples [60]. During the recovery process, the deformed grains reduced their stored energy through the removal or rearrangement of defects in their crystal structure [64]. In PFB samples, some defects occurred during manufacturing which may initiate a recovery process. Dislocations were rearranged during recovery and subsequently transformed into LABs (low-angle boundaries), leading to the formation of sub-grains [61]. Heating samples at 1150 °C even for one hour causes the microstructure to undergo homogenization. Anisotropic columnar grains, dendritic structures, and sub-grains for samples with all build angles were transformed into uniform equiaxed grains with no apparent internal disorientation. The residual strain inside the grains was relieved [63] as a result of thermally induced recrystallization, which is likely driven by the strain energy stored in the microstructure by differential thermal contractions during the PBF process [65,66]. High dislocation densities are present at the boundaries of the cellular dendritic structures, observed in the finished PBF products, which result in stored energy in these regions. The structure of the recrystallized grains depend on the level of accumulated (residual) stress in the region [65]. Due to recrystallization, the heat-treated (1150 °C) samples display new equiaxed grains containing a number of Σ3 annealing twin boundaries with new crystal orientations and the residual plastic strain inside the grains was drastically relieved. This process results in grains with uniformly-distributed crystal orientations and a homogenous isotropic microstructure [61].

It should be emphasized that the products manufactured by casting also have some defects which significantly affect alloy ductility. Thermal processing has been commonly used in casted Co–Cr alloys to improve their final properties. In addition to structure homogenization, the formation of metal carbides is known to influence Co-based alloy (comparable to standard specification ASTM F75) performance [67,68]. Carbides inside the grains strengthen the alloy matrix, acting as obstacles in dislocations while they prevent grain boundary sliding and migration at the grain boundaries [62,69]. The main factors which influence the size and amount of carbides are the heat-treatment temperature and the cooling rates. The dissolution of carbides into matrix occurs above 1200 °C [70]. After this heat treatment, slow cooling rates promote the development of a lamellar eutectic structure with carbides at the grain boundaries [71]. The use of strong cooling (e.g., water quenching from heat treatment temperature) causes the formation of fine primary carbide which are more homogenously distributed in the matrix [72]. By increasing the cooling rate, the size of the carbides formed decreases [73]. Moreover, in PBF materials subjected to heat treatment, there are precipitations that occur both in the grains and on their boundaries. The amount, size and distribution of the precipitations will depend mainly on the temperature, heating time and cooling method. Furnace cooling contributes to the formation of larger precipitates at the grain boundary regions. Water quenching promotes the formation of smaller precipitates and carbide dissolution into the matrix [74,75].

Increasing the heat-treatment temperature decreased by 0.2% the yield strength, ultimate tensile strength and Vickers hardness of the CoCr alloy specimens but enhanced the ductility. Recovery processes, which occurred in the heat treatment under 1050 °C, resulted in a reduced dislocation density and the volume fraction of the ε phase. This explains the reduction in material strength and an increase in the ductility. Elongation is an important factor, especially in the removal partial dentures. Denture parts characterized by low elongation can fracture more easily than those with high elongation. The reduction of the ε phase in alloys results in sample elongation. Considering the characteristics of loads that occur in the oral environment, one of the key properties of prosthetic components is fatigue resistance. The fatigue strength of SLM samples was improved after heat treatment at 1150 °C [63]. However, in one study, controls with 90° building angulations exhibited longer fatigue life than the heat-treated groups [45]. It was explained due to fine cellular or columnar dendritic structures which have a function as obstacles for the dislocation motion. Samples with 0° and 45° building angulations showed a lower fatigue life than the heat-treated groups due to the occurrence of molten pool boundaries and Schmid factor [45]. Extended heat treatment (six hours) resulted in large precipitates (large blocky carbides formed at the grain boundaries) and a thick oxide layer which decreased fatigue strength [45]. The optimization of the heat-treatment conditions for improving the mechanical properties remains a major challenge in SLM.

### 6.2. Surface Finishing 

One significant challenge to the commercialization of 3D printed dental products is the variable surface finish, which can compromise the aesthetic, technical function and the achievable tolerances. The surface roughness of PBF products is dependent on the process parameters, the position and orientation to the building direction and the choice of the supports [18,28]. Traditional polishing methods (mechanical, ultrasonic, ion-beam, and chemical) have some limitations in processing PBF parts. Recent studies have focused on surface modification using lasers to satisfy strict surface requirements in medical applications. The surface quality of additive manufactured parts can be significantly improve by laser polishing [76,77]. Additionally, some studies show that the surface microstructure and corrosion resistance was improved by controlling the hexagonal close packed structures and the formed oxides [78].

The sterilization of medical products is a vital step before their clinical use. It can be managed by using irradiation (Gamma and E-beam), plasma and chemicals (peracetic acid). These methods are efficient but expensive. Dry heat and steam (autoclave) sterilization both proved to be efficient in the case of dental implants produced by SLM (even with a porous structure) [79].

## 7. Summary 

Additive manufacturing processes have opened new possibilities, allowing the production of complex components, individually tailored to the patient. In dentistry, SLM is the most popular of all powder bed fusion technologies. Seemingly, this method seems to be less complicated than casting. However, the CAD–CAM specialist should have the knowledge to choose the right process parameters as well as post-processing methods. Without this ability, the resulting product may not be suitable for reliable use in practical applications.

The following conclusions can be drawn from the literature review:

1. PBF-manufactured parts are characterized by an anisotropic γ-phase (face-centered cubic (fcc)) and ε-phase (hexagonal close-packed (hcp)). The microstructure, roughness and properties of the samples depend on build orientation. Parts built at 0° were characterized by the worst mechanical properties.
2. Basic parameters, such as the laser power, scanning speed, laser beam size and layer thickness, are connected and they influence the density of the melted material. These parameters determine the material properties. In the SLM processes, energy input values above 0.36 J/s result in reduced sample density. The optimized range of laser energy density (LED) for Co_28_Cr_6_Mo SLM parts is 150–200 J mm^−3^.
3. Post-processing heat treatment is considered necessary to ensure the reliable use of PFB-built parts in practical applications. Heat-treatment up to 1050 °C (even for six hours) is insufficient to eliminate anisotropy and residual stress. As heat-treatment temperatures increase from 750 °C to 1050 °C, homogenization also increases, compared with the as-built samples. Heating samples at 1150 °C for one hour causes the total homogenization of the sample’s microstructure.
4. Optimization of the heat-treatment conditions for improving the mechanical properties (especially fatigue properties) remains a major challenge in SLM.
5. Surface finishing of PFB-built parts is necessary.

## Figures and Tables

**Table 1:** Computer-aided design/computer-aided manufacturing (CAD/CAM) processes currently upgraded into dentistry.

Process	Technology	Ref.
Digital impression	It is a non-invasive method to obtain a virtual model of the hard and soft tissue of the patient’s oral cavity. This involves the use of an intraoral scanner which records a series of snapshots of the oral cavity of the patient.	[22]
Prosthetic designing	Scanner data are evaluated and processed by the dental laboratory using special software. The digital prosthetic model is individually tailored to the patient.	[22]
Manufacturing process	Milling (subtractive manufacturing): a prepared prosthetic design is mechanically cut from a metal block. This process is controlled by software. Milling units in the last decade were optimized—large angulations of the fourth and fifth axes (>30 degrees), dry or wet grinding.	[5]
	Sintering (subtractive manufacturing): technology developed by Amann Girrbach. The prepared prosthetic is mechanically cut from metal blanks with a wax-like texture. The process is controlled by software. The blocks are made from unsintered metal powder held together by a binder. After the milling process, the structure is subjected to sintering in a special furnace.	
	Powder bed fusion (PBF, additive manufacturing): the prepared prosthetic is formed by the thermal consolidation of a metal powder (layer by layer). The process is controlled by software. Selective laser sintering (SLS), selective laser melting (SLM) and electron beam melting (EBM) are three methods classified as PBF.	

**Table 2:** Brands and chemical composition of CoCr alloys for dental (DA) and medical (MA) applications. Data from reference [19].

Brand	PBF System	Chemical Composition (wt.%)
EOS CoCr SP2 (DA)	DMLS (direct metal laser solidification) = SLM	Co: 63.8; Cr: 24.7; Mo: 5.1; W: 5.4; Si: 1.0; Fe: ≤0.50; Mn: ≤0.10 Free of Ni, Be, Cd and Pb
EOS CoCr MP1(MA)	DMLS (direct metal laser solidification) = SLM	Co: 60–65; Cr: 26–30; Mo: 5–7; Si: ≤1.0; Mn: ≤1.0; Fe: ≤0.75; C: ≤0.16; Ni: ≤0.10
Renishaw CoCr DG1(DA)	SLM	Co: 63.9; Cr: 24.7; Mo: 5.0; W: 5.4; Si: 1.0 Free of Ni, Be, Cd and Pb
SLM Solutions MediDent (DA)	SLM	Co: Balance; Cr: 22.7–26.7; Mo: 4–6; W: 4.4–6.4; Si: 2; Fe: 0.5; Mn: 0.10; C: 0.02; Ni: 0.10; B, S: 0.10
SLM Solutions CoCr28Mo6 (MA)	SLM	Co: Balance; Cr: 27–30; Mo: 5–7; W: 0.20; Si: 1; Al: 0.10; Fe: 0.75; Mn: 1; C: 0.35; N: 0.25; Ni: 0.5 Ti: 0.1; B, S: 0.01
3D systems LaserForm CoCr(A,B,C) ASTM F75(DA and MA)	SLM	Co Bal., Cr 28.00–30.00, Mo 5.00–6.00, Ni 0.00–0.10, Fe 0.00–0.50, C 0.00–0.02, Si 0.00–1.00, Mn 0.00–1.00, Cd 0.00–0.02, Be 0.00–0.02, Pb 0.00–0.02
Concept Laser Remanium star CL	DMLM (direct metal laser melting)	Co: 60.5; Cr: 28; W: 9; Si: 1.5; Other elements <1: Mn, N, Nb, Fe. free from nickel, beryllium and gallium
BEGO Wirobond C+	SLM	Co: 63.9; Cr: 24.7; W: 5.4; Mo: 5.0; Si: <1
Scheftner Starbond COS	SLM	Co: 59; Cr: 25; W: 9.5; Mo: 3.5; Si: 1; C,Fe,Mn,N: <1%
Scheftner Starbond Easy 30	SLM	Co: 61; Cr: 27.5; W: 8.5; Si: 1,6; C,Fe,Mn: <1

**Table 3:** Physical and mechanical properties of 3D printed CoCr (after stress relief) provided by their manufacturers after heat treatment. NA: Not available. Data from Reference [19].

Property	OS SP2	EOS MP1	Renisaw DG1	SLM MediDent	SLM CoCr28Mo6	LaserForm CoCr (B)	Concept Laser Remanium Star CL	BEGO Wirobond C+	Scheftner Starbond COS	Scheftner Starbond Easy 30
Density (g/cm^3^)	8.5	8.3	8.3	NA	NA	8.3	8.6	8.6	8.8	8.5
Tensile strength (MPa)	1350	1100	1076	1415	1215	1445 +/− 50	1030	1315	990–1250	1090
Elongation at break (%)	3	Min. 20	2.7	4	21	34 +/− 6	10		2–10	15
Young’s Modulus (GPa)	approx. 200	200	224	245	205	230 +/− 40	230	215	195–200	225
Hardness	420 HV	approx. 35–45 HRC	430 HV	NA/375 HV as built	385 HV	26 +/− 5 Rockwell C	NA	NA	345–490 HV 10	425 HV 10
Coefficient of thermal expansion	14.3 × 10^−6^ m/m °C	13.6 × 10^−6^ m/m °C–15.1 × 10^−6^ m/m °C	14.1	NA	NA	14	14.1 (500 °C)	14.3 (RT-500 °C) 14.5 (RT-600 °C)	14.4 (600 °C)	14.5 (500 °C) 14.7(600 °C)
Melting interval (°C)	1410–1450	1350–1430	1375–1405	NA	NA	1350–1430	1320–1420	1380–1420	1305–1400	1310–1410

**Table 4:** Heat treatment procedures of 3D printed CoCr.

Procedure	Note	References
Under an argon atmosphere. 1. Heat furnace from room temperature to 450 °C in 60 min and hold at this temperature for 45 min. 3. Heat furnace to 750 °C in 45 min and hold at this temperature for 60 min (holding temperature and time tolerance inside the box: 740 °C +/− 10 °C, 60 minutes +/− 20 min). 5. Switch off the heating. 6. When temperature has dropped down to approx. 600 °C, open the furnace door. 7. When furnace has cooled down to approx. 300 °C, remove the protective gas box from furnace and shut down the argon flow.	Instructions for use material: EOS CobaltChrome SP2. Longer holding time, utilization of higher stress-relieving temperature or forced cooling may lead to increased brittleness of restorations.	[56]
Post-process stress relieving at 1150 °C for six hours.	Instructions for use material: EOS CoCr MP1 for medical applications.	[57]
Under an argon atmosphere. Hold at 750 °C temperature for 60 min and allowed to cool naturally.	Instructions for use Renishaw CoCr DG1.	[58]
I. First heating process 1. Heat furnace from room temperature to 500 °C (a ramp rate of 8 °C/min) and hold at this temperature for 45 min. 2. Heat furnace to 880 °C in 60 min, and hold at this temperature for 60 min. 3. Switch off the heating. 4. When temperature has dropped down to approx. 600 °C, open the furnace door. 5. Remove specimens from the furnace when the temperature has dropped to 300 °C. II. Second heating process 1. Heat furnace from room temperature to 1100 °C (a ramp rate of 8 °C/min) and hold at this temperature for 30 min. 2. Switch off the heating. 3. When temperature has dropped down to approx. 600 °C, open the furnace door. 4. Remove specimens from the furnace when the temperature has dropped to 300 °C.	Heat treated SLM-fabricated samples (at 880 °C and 1100 °C) exhibited mechanical properties that exceeded the minimum requirements according to ISO standard (ISO 22674:2016.8). The heat treatment at 1100 °C was more efficient for relieving residual stress.	[55]
1. Heat furnace from room temperature to 1150 °C and hold at this temperature for 60 min. 2. Cool samples in water (water quenching) 3. Half of the samples were additionally subjected to isothermal heat treatment at 800 °C for four hours 4. Cool samples in water (water quenching)	The elimination of dendritic microstructure and micro-segregation was achieved after heat treatment at 1150 °C. Increased tensile strength and yield stress were achieved by isothermal heat treatment at 800 °C and water quenching due to a diffusely formed hcp ε phase, finely distributed in the fcc γ matrix. Crack initiation and propagation occur preferentially at the ε martensite.	[59]
Under an argon atmosphere. 1. Heat furnace from room temperature to 750, 900, 1050, or 1150 °C (a ramp rate of 60 °C/min) and hold at the specified temperature for six hours. 2. Switch off the heating. 3. When temperature has dropped down to approx. 300 °C, open the furnace door.	Heat treatment at temperatures below 1050 °C was insufficient to eliminate the anisotropic columnar grains, fiber texture and residual stress. Recrystallization via heat treatment at 1150 °C homogenizes the microstructure, eliminate residual stress and enables reduction of the anisotropic mechanical properties. Increasing the heat-treatment temperature from 750 °C to 1150 °C increased the ductility of the alloy and decreased its 0.2% offset yield strength and Vickers hardness.	[60,61]
The three heat treatment cycles were used: I. One hour at 1220 °C; II. Four hours preheating at 815 °C and then two hours at 1220 °C; III. Four hours at 1220 °C. Rapid quenching in water at room temperature was done at the end of preheating and solution treatment.	After heat treatment, both the ultimate tensile strength and yield strength of SLM CoCrMo decreased due to the homogenization of microstructure and texture. The microstructure analysis concluded carbides formation.	[62]
Under an argon atmosphere. 1. Heat furnace from room temperature to 600 °C (a ramp rate of 10 °C/min) and hold at this temperature for 30 minutes. 2. Heat furnace to 1150 °C and hold at this temperature for one or six hours. 3. After one or six hours switch off the heating. 4. Cool samples slowly to room temperature in a furnace.	Heat treatment at 1150 °C for one and six hours allowed the homogenization of microstructure and texture. Higher fatigue strength was observed after 1 h treatment compared with control groups (as built 0° and 45° building angulations). Six hours heat treatment created large precipitates and a thick oxide layer associated with a decrease in fatigue strength.	[45,63]
